# The Hospital Egg: Merchants' Views

**Published:** 1915-07-24

**Authors:** 


*To the Editor of* The Hospital.

### The Hospital Egg: Merchants' Views.

*To the Editor of* The Hospital.

Sir,—May we be allowed to venture an opinion on the all-important subject of the hospital egg, as we have had some experience on this matter? We do not believe that prices will soar as high as those mentioned by Mr. Alexander. True, if we all had to rely upon English producers that might happen, but the Irish people have risen to the great occasion in a manner the English have not even thought of, and we do not anticipate such famine prices, unless, of course, something extraordinary happens. The great difficulty here is that the average producer thinks in dozens, or, perhaps, odd hundreds, whereas the hospital consumption runs up to thousands per week each, so emphasising the great necessity for merchants to equalise the demands by making, as we have to do, very adequate preparations to obtain ample supplies of really new-laid eggs all the year round, mid-December as well as the plentiful spring months.

We do not mind admitting that we have contracts running at very much less than the price mentioned, being under contract to supply many military and ordinary hospitals, and we ought to have some sort of an idea as to probable costs.

As our business is solely in eggs, we have to study all phases of this question, and we unhesitatingly say that pickled or preserved eggs are not really satisfactory for use in hospitals. All the dodging and coaxing in the world will not make a stale egg into a new-laid. We know the Danes preserve lots of eggs, and so do some Irish firms, but hospital staffs will not find it profitable or even advantageous. After all, preserved or pickled or cold-stored eggs are fit for nothing better than cooking. To poach, an egg must be really new-laid. New-laids are alone fit for egg-cup use.

There will be no famine in what are popularly known as "fresh" eggs. Your advice in the foot-note after Mr. Alexander's letter is very sound, and we do advise those having the important task of buying for hospitals to insist upon new-laid eggs, at least for all purposes other than merely cooking, and to insist on those being tested and guaranteed. That at least is what we do, and our numerous customers over an area up to 130 miles from London have not much fear of famine prices or inability to buy new-laid eggs. Last year we sent thousands over to the base hospitals in France, and when the National Egg Collection supplies fall off we hope to be doing so again, and have assured the authorities of our ability to provide very large quantities, and hope to take on several more hospitals.—Yours faithfully,

Wilkinson Bros.,

Wholesale New-laid Egg Merchants.

37 Wilton Place, Knightsbridge, S.W.

July 17, 1915.

